# Mitochondrial-Associated Cell Death Mechanisms Are Reset to an Embryonic-Like State in Aged Donor-Derived iPS Cells Harboring Chromosomal Aberrations

**DOI:** 10.1371/journal.pone.0027352

**Published:** 2011-11-14

**Authors:** Alessandro Prigione, Amir M. Hossini, Björn Lichtner, Akdes Serin, Beatrix Fauler, Matthias Megges, Rudi Lurz, Hans Lehrach, Eugenia Makrantonaki, Christos C. Zouboulis, James Adjaye

**Affiliations:** 1 Molecular Embryology and Aging Group, Department of Vertebrate Genomics, Max Planck Institute for Molecular Genetics, Berlin, Germany; 2 Departments of Dermatology, Venereology, Allergology and Immunology, Dessau Medical Center, Dessau, Germany; 3 Department of Computational Molecular Biology, Max Planck Institute for Molecular Genetics, Berlin, Germany; 4 Electron Microscopy Group, Max Planck Institute for Molecular Genetics, Berlin, Germany; 5 Institute of Clinical Pharmacology and Toxicology, Charité University Medicine, Berlin, Germany; 6 The Stem Cell Unit, Department of Anatomy, College of Medicine, King Saud University, Riyadh, Saudi Arabia; National Institute on Aging Intramural Research Program, United States of America

## Abstract

Somatic cells reprogrammed into induced pluripotent stem cells (iPSCs) acquire features of human embryonic stem cells (hESCs) and thus represent a promising source for cellular therapy of debilitating diseases, such as age-related disorders. However, reprogrammed cell lines have been found to harbor various genomic alterations. In addition, we recently discovered that the mitochondrial DNA of human fibroblasts also undergoes random mutational events upon reprogramming. Aged somatic cells might possess high susceptibility to nuclear and mitochondrial genome instability. Hence, concerns over the oncogenic potential of reprogrammed cells due to the lack of genomic integrity may hinder the applicability of iPSC-based therapies for age-associated conditions. Here, we investigated whether aged reprogrammed cells harboring chromosomal abnormalities show resistance to apoptotic cell death or mitochondrial-associated oxidative stress, both hallmarks of cancer transformation. Four iPSC lines were generated from dermal fibroblasts derived from an 84-year-old woman, representing the oldest human donor so far reprogrammed to pluripotency. Despite the presence of karyotype aberrations, all aged-iPSCs were able to differentiate into neurons, re-establish telomerase activity, and reconfigure mitochondrial ultra-structure and functionality to a hESC-like state. Importantly, aged-iPSCs exhibited high sensitivity to drug-induced apoptosis and low levels of oxidative stress and DNA damage, in a similar fashion as iPSCs derived from young donors and hESCs. Thus, the occurrence of chromosomal abnormalities within aged reprogrammed cells might not be sufficient to over-ride the cellular surveillance machinery and induce malignant transformation through the alteration of mitochondrial-associated cell death. Taken together, we unveiled that cellular reprogramming is capable of reversing aging-related features in somatic cells from a very old subject, despite the presence of genomic alterations. Nevertheless, we believe it will be essential to develop reprogramming protocols capable of safeguarding the integrity of the genome of aged somatic cells, before employing iPSC-based therapy for age-associated disorders.

## Introduction

A novel strategy to derive pluripotent stem cells from adult somatic tissue, called cellular reprogramming, recently revolutionized the field of regenerative medicine [1], [2]. In comparison to human embryonic stem cells (hESCs), induced pluripotent stem cells (iPSCs) exhibit two major advantages: (i) their generation is not hampered by the ethical issues commonly associated with blastocyst-derived stem cells and (ii) they represent individual-specific isogenic cells. Thus, iPSCs hold the potentiality to be used for personalized drug-screening [3], and patient-tailored regenerative therapies without the risk of immune rejections [4]. The iPSC technology is of particular interest in the context of age-associated disorders, such as Alzheimer's and Parkinson's disease, which affect a growing number of people and currently lack efficacious treatments.

Unfortunately, loss of genome integrity has been observed within hESCs and iPSCs by different groups [5], [6]. In particular, the reprogramming process has been found linked to a high mutation rate [7], [8], [9]. Additionally, we recently unveiled that the mitochondrial genome of human fibroblasts also undergoes random mutational events upon the induction of pluripotency [10]. Aged somatic cells might be even more susceptible to nuclear and mitochondrial genome instability, due to an aging-related increase in oxidative DNA damage [11]. Genes involved in genome integrity have been shown to be repressed with advancing age [12] and maternal aging positively correlates with the rate of aneuploidy [13]. Accordingly, chromosomal aberrations have been recently observed in iPSC lines obtained from elderly individuals [14].

The reprogramming-associated genomic alterations may exert a tumorigenic effect. Indeed, deletions of tumor-suppressor genes have been detected during reprogramming, while duplications of oncogenic genes have been observed upon extended culture [5], [6]. Moreover, point mutations within cancer-related genes were identified within iPSC lines derived with various reprogramming methods [7], [8], [9]. Nonetheless, no particular tumorigenic mechanism has yet been identified as consistently functionally implicated during iPSCs generation or upon their adaptation in culture. In accordance, genomic aberrations within iPSCs did not alter their cellular functionality [7], [8], [9], [14] and mitochondrial DNA (mtDNA) modifications did not affect the reprogramming-associated re-modulation of energy metabolism [10]. Hence, it is essential to understand the biological significance of reprogramming-induced genetic alterations and to determine whether their presence can truly enhance the oncogenic potential of individual iPSC lines.

Besides aneuploidy, an important hallmark of cancer cells is their resistance to apoptosis [15]. This ability to escape cell death is in fact essential to achieve unlimited and uncontrolled proliferation. Recent data has demonstrated that activation of apoptosis has a mediatory role in the induction of pluripotency, as two main proteases involved in programmed cell death, Caspases 3 and 8, are activated by OCT4 [16]. Mouse and human ESCs possess profound susceptibility to apoptosis-inducing DNA damaging agents [17], [18]. Human iPSCs have also been found to exhibit hypersensitivity to DNA damaging agents following γ-irradiation, which results in rapid induction of apoptosis [19]. Thus, it is important to examine whether genomic abnormalities within pluripotent stem cells might induce oncogenic transformation by altering the susceptibility to apoptosis, a mechanism essential for safe-guarding against uncontrolled cancer-like proliferation.

Cell death mechanisms are tightly regulated by mitochondria, which, besides being the cellular powerhouse, are the active site of apoptotic induction and reactive oxygen species (ROS) generation [20]. Over time, the presence of mitochondrial dysfunctions may exert cellular detrimental effects leading to imbalances in energy production, modulation of programmed cell death, or redox maintenance. In fact, oxidative stress induces the accumulation of mutational damage within mtDNA [21], which in turn can cause multiple cellular dysfunctions [22], [23]. We and others have recently demonstrated that somatic mitochondria undergo a complex remodeling upon reprogramming, as they adapt their morphology and functionality to attain the pluripotent state [24], [25], [26]. Therefore, iPSC generation modulates mitochondrial-related pathways to dictate the acquirement of a hESC-like redox homeostasis.

In the present work, we investigated whether the presence of chromosomal aberrations may be linked to tumorigenicity by an alteration of mitochondrial-associated cell death mechanisms. This was conducted in skin-derived cells from very old individuals, which might harbor alterations in signaling pathways related to mitochondria and oxidative stress [27], and thus may be more prone to genomic instability. Four iPSC lines were generated from dermal fibroblasts obtained from an 84-year-old woman, which to our knowledge represent the most aged human donor cells reprogrammed to pluripotency. All lines exhibited chromosomal abnormalities that were not observed in any of the iPSC lines we have derived in our laboratory from young individuals [10], [26], [28], [29]. These aberrations included the enrichment of pluripotency and cancer-associated genes. Nevertheless, the presence of karyotype variations did not affect the ability of aged-iPSC lines to re-establish telomerase activity, differentiate into neuronal cells *in vitro*, or induce X-chromosome reactivation. Importantly, mitochondria ultra-structural morphology and functionality as well as mitochondrial modulation of programmed cell death and ROS generation were also reset to an embryonic-like state regardless of chromosomal abnormalities. Both young and aged donor-derived iPSCs exhibited a similar hESC-like hypersensitivity to apoptosis and low levels of ROS and DNA damage. Overall, our data suggest that the presence of chromosomal aberrations within aged reprogrammed cells may not exert a detrimental effect by inducing a reduction of cell death or an increased in cellular oxidative stress, which are necessary mechanisms for tumor development and progression.

## Results

### Generation and characterization of aged donor-derived iPSCs

**Figure 1:**
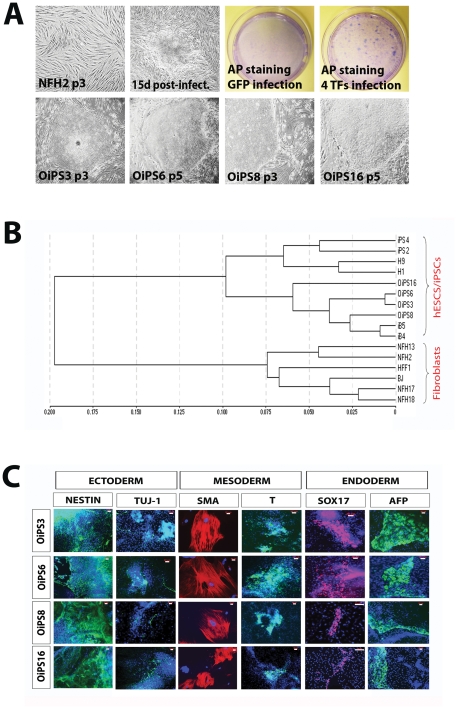
Derivation and characterization of four iPSC lines from an 84-year-old woman. (**A**) NFH2 dermal fibroblasts were obtained from an 84-year-old woman and transduced using retroviruses containing the four transcription factors (TFs) OCT4, KLF4, SOX2 and c-MYC. Four weeks later, hESC-like colonies were stained for alkaline phosphatase (AP) activity then manually picked for further characterization. Four distinct iPSC lines were established: OiPS3, OiPS6, OiPS8, and OiPS16. (**B**) Hierarchical clustering of all fibroblast cells and pluripotent stem cells. NFH2-derived iPSC lines acquired a pluripotent stem cell-like transcriptional signature and clustered far apart from the parental NFH2 fibroblasts and from other fibroblast cells. (**C**) Embryoid body (EB)-based *in vitro* differentiation of the four NFH2-derived iPSC lines. All differentiated NFH2-iPSC lines expressed marker proteins specific to the three germ layers: ectoderm (NESTIN and TUJ-1), mesoderm (SMOOTH MUSCLE ACTIN (SMA) and BRACHYURY (T)), and endoderm (SOX17 and ALPHA FETO PROTEIN (AFP)). Scale bars, 20 µm.

Aged donor-derived dermal fibroblasts were obtained from an 84-year-old woman (NFH2) affected by age-associated medical conditions, including late-onset diabetes type II, hypertension, and renal failure (Table S1). NFH2-iPSCs were generated by retroviral transduction of the Yamanaka cocktail (OCT4, KLF4, SOX2, and c-MYC) [1]. Four iPSC lines were established and fully characterized: OiPS3, OiPS6, OiPS8, and OiPS16 (Figure 1A). All lines exhibited hESC-like morphology and growth rate, alkaline phosphatase (AP) activity, expression of pluripotency-associated markers NANOG, SSEA4, TRA-1-60, TRA-1-81, and the genetic fingerprinting pattern of NFH2 parental fibroblasts (Figure S1). The four aged-iPSC lines were compared to two hESCs (H1 and H9) and to four iPSC lines previously generated from two neonatal foreskin fibroblasts: iPS2 and iPS4 from HFF1 fibroblasts and iB4 and iB5 from BJ fibroblasts [26]. All aged-iPSC lines acquired a gene expression signature similar to that of hESCs (Pearson correlation value: 0.89) and young donor-derived iPSCs (Pearson correlation value: 0.82) and distinct from that of the parental NFH2 fibroblasts (Pearson correlation value: 0.67) (Figure 1B, Figure S2).

The differentiation potential of aged donor-derived iPSCs was tested both *in vitro* and *in vivo*. All lines could successfully differentiate *in vitro* into all three embryonic germ layers, as detected by the expression of marker proteins specific for ectoderm, NESTIN and TUJ-1, for mesoderm, SMOOTH MUSCLE ACTIN (SMA) and BRACHYURY (T), and for endoderm, SOX17 and ALPHA FETO PROTEIN (AFP) (Figure 1C). Two aged-iPSC lines (OiPS6 and OiPS16) were able to generate teratomas containing derivatives of all three germ layers. However, the presence of known endoderm-associated structures could not be identified within teratoma samples of the remaining two iPSC lines (OiPS3 and OiPS8) (Figure S3). Additional studies are ongoing to determine whether these two lines conclusively exhibit a real impairment toward endoderm differentiation *in vivo* or the observation is potentially due to the deficiencies inherent to the current teratoma assays [30].

### Chromosomal aberrations in aged reprogrammed cells

**Figure 2:**
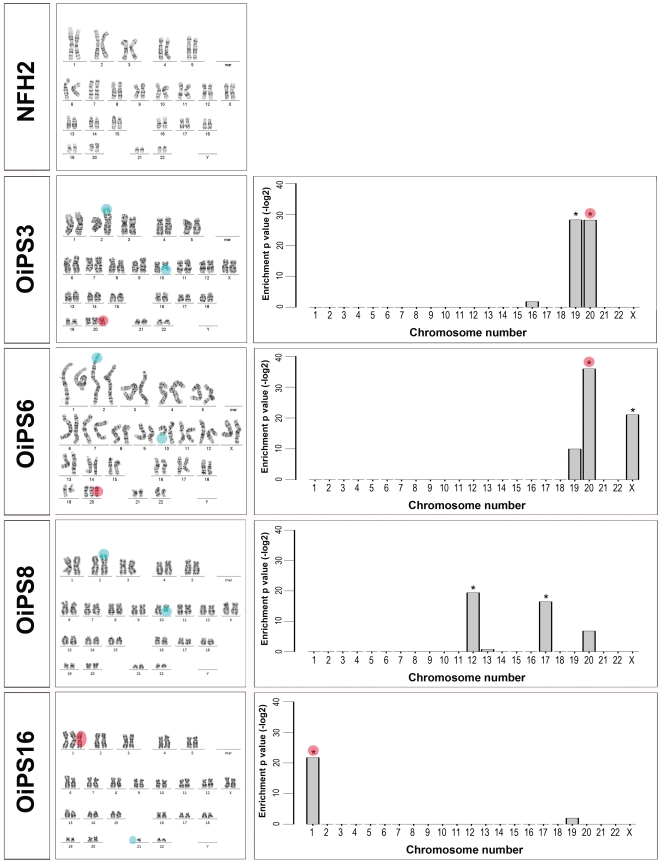
Chromosomal aberrations in aged reprogrammed cells. Left panel, karyotype analysis of the parental NFH2 fibroblast cells and NFH2-derived iPSC lines. NFH2 was karyotypically normal, while all NFH2-iPSC lines exhibited chromosomal aberrations (red circles and light blue circles). Right panel, transcriptional profiling-based analysis of chromosomal integrity. Expression values of single genes in NFH2-iPSC lines were compared to the parental NFH2 fibroblasts and considered over-expressed when fold-change>2 and Bonferroni corrected p-values <10^−4^. The analysis confirmed significant enrichment in all cases in which karyotyping showed trisomic transformation (red circles). Additional significant enrichments could be observed in NFH2-iPSCs in chromosomes that appeared normal upon karyotyping. Chromosomal translocations (10 to 2) in OiPS3, OiPS6, OiPS8 and chromosomal 1 loss in OiPS16 could not be confirmed by gene expression-based chromosomal enrichment analysis (light blue circles).

Genetic integrity of the four aged donor-derived iPSC lines was first assessed by karyotyping. The analysis revealed the presence of chromosomal abnormalities that were not detected in the parental NFH2 fibroblast cells (Figure 2). These results were unexpected, since all young donor-derived iPSC lines (from neonate foreskin fibroblasts, from amniotic fluid cells, and from chorionic villi cells) previously established in our laboratory bore no karyotype abnormalities [10], [26], [29], [31]. The aberrations detected in aged-iPSCs included trisomy of chromosome 20 (in OiPS3 and OiPS6), translocation between chromosome 2 and 10 (in OiPS3, OiPS6, and OiPS8), trisomy of chromosome 1 (in OiPS16) and loss of chromosome 21 (in OiPS16). OiPS16 exhibited a mixed population, where a portion of cells harbored a normal karyotype, while the remaining acquired chromosomal abnormalities (Figure 2, red circles and light blue circles).

To investigate genome instability in more detail, we analyzed chromosomal integrity using a recently published gene expression-based approach [6]. Transcriptome data of young and old donor-derived iPSCs were compared to their corresponding parental fibroblast cells. For hESCs, the expression values were presented with reference to the median of all pluripotent stem cells included in the dataset [6]. Significant chromosomal enrichment (Bonferroni corrected p-values <10^−4^) could be confirmed in all trisomy cases, namely chromosome 20 in OiPS3 and OiPS6, and chromosome 1 in OiPS16 (Figure 2, red circles). In addition, significant enrichment could be identified in chromosomes that were found complete upon karyotyping, including chromosome 12 in H1 and OiPS8, chromosome 16 in iPS4 and iB5, chromosomes 17 in OiPS8, chromosome 9 in OiPS3, and chromosome X in OiPS6 (Figure 2 and Figure S4). Nevertheless, gene expression-based analysis failed to confirm the 10 to 2 translocation (found in lines OiPS3, OiPS6, and OiPS8) and the 21 loss detected in a sub-population of OiPS16 (Figure 2, light blue circles). Hence, this approach might not be appropriate to detect genomic changes occurring in short chromosomes within a mixed population, as already pointed out [5], or to identify chromosomal translocations. Indeed, although a portion of chromosome 10 was physically located on chromosome 2, the genes of the misplaced chromosomal area were still assigned to chromosome 10, and thus considered unaffected by gene expression-based analysis.

**Figure 3:**
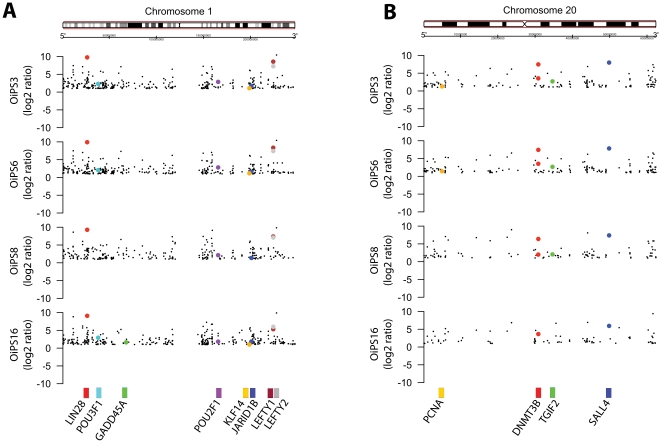
Enrichment of pluripotency and tumorigenicity-associated genes within chromosomes 1 and 20 in aged-iPSCs. (**A**) Moving average plot analysis of chromosome 1. Expression values in NFH2-iPSC lines were compared to the parental NFH2 fibroblasts and considered over-expressed or under-expressed when fold-change>2. Each dot represents a probeset encompassing an individual gene. Highlighted in different colors are probeset for genes whose function has been associated with pluripotency, reprogramming, proliferation, or tumorigenicity. (**B**) Moving average plot analysis of chromosome 20. Moving average plots were produced using R/Bioconductor *package GenomeGraphs*.

Recent data suggest that certain types of aneuploidies within hESCs and iPSCs may induce a proliferative advantage by increasing the expression pluripotency-associated genes [5], [6]. Interestingly, chromosome 1 and 20, which exhibited a trisomic transformation within our aged-iPSC lines, were among the most frequently affected chromosomes harboring chromosomal gain [6] and copy number variation [5] in hESCs and iPSCs. Moving average plot analysis revealed that genes such as *LIN28*, *LEFTY1* and *LEFTY2* on chromosome 1 (Figure 3A) and *DNMT3B* and *SALL4* on chromosome 20 (Figure 3B), known to be involved in the regulation of pluripotency, were indeed significantly enriched in NFH2-iPSCs compared to the parental NFH2 fibroblasts. Up-regulated genes comprised members of transcription factor families associated with reprogramming, such as the POU family (*POU2F1* and *POU3F1*) and the Kruppel-like family (*KLF14*) (Figure 3). Moreover, genes regulating proliferation and linked to tumorigenicity, such as *JARID1B*, *PCNA*, *TGIF2*, and the p53 target gene *GADD45A,* were also detected among the genes within chromosome 1 and 20 enriched in NFH2-iPSCs compared to NFH2 fibroblasts (Figure 3). The complete list of significantly altered genes in all our pluripotent stem cell lines is reported in Table S2.

### Chromosomal aberrations within aged-iPSCs do not affect neuronal differentiation, X-chromosome inactivation (XCI), and telomerase activity

**Figure 4:**
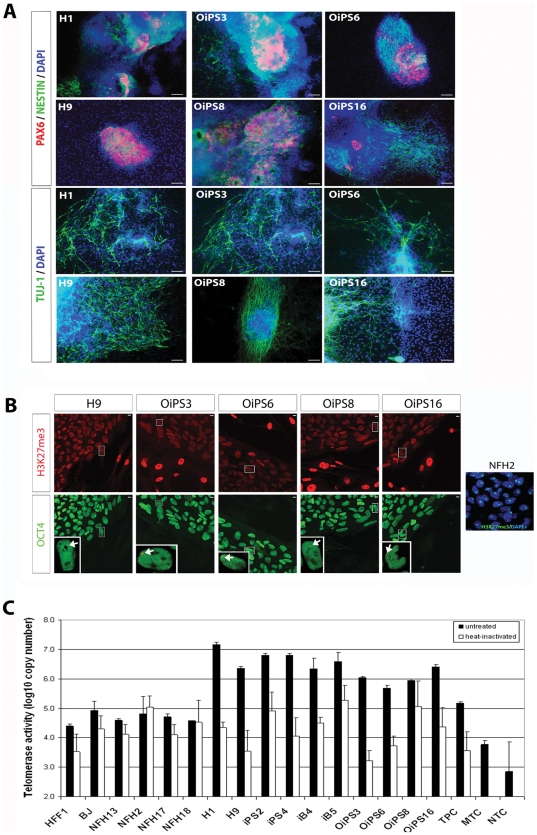
Neuronal differentiation, X-chromosome inactivation, and telomerase activity in aged donor-derived iPSCs. (**A**) Neuronal cells were derived by the combined treatment of transforming growth factor β(TGFβ) receptor inhibitor and MEK1/2 inhibitor. All aged-iPSC lines generated neuronal-like cells expressing PAX6, NESTIN, and TUJ-1, in a similar fashion to the hESC lines H1 and H9. Scale bars, 10 µm. (**B**) X-chromosome inactivation (XCI) was assayed by H3K27me3 immunostaining. OCT4 staining was used to distinguish undifferentiated pluripotent stem cells from MEFs. NFH2 fibroblasts clearly exhibited inactive X-chromosomes (Xi), while variable XCI status was observed in both H9 and NFH2-derived iPSC lines. Single female pluripotent cells expressing Xi are indicated by the white arrows in the bottom inserts. Scale bars, 10 µm. (**C**) Telomerase activity was low in fibroblasts from neonates (HFF1 and BJ) and from adult individuals of various ages (NFH13-55y, NFH2-84y, NFH17-80y, and NFH18-80y). The level of activity was similarly elevated in all pluripotent stem cells, including hESCs (H1 and H9), HFF1-iPSCs (iPS2 and iPS4), BJ-iPSCs (iB4 and iB5) and NFH2-iPSCs (OiPS3, OiPS6, OiPS8, and OiPS16). Heat inactivation was performed to show the different enzymatic activity between the active and inactive state. TPC, telomerase positive extract control (100 ng of positive control cell protein extract provided by the kit); MTC, minus telomerase control (cell sample substituted by CHAPS Lysis Buffer); NTC, no template Control (cell sample substituted by water). Error bars indicate standard deviation.

In order to test whether chromosomal aberrations could hamper differentiation capabilities, the four aged donor-derived iPSC lines were subjected to direct neuronal differentiation. Neuronal cells were generated following a recently published adherent protocol, which requires the exposure to TGF-β receptor inhibitor (SB) and MEK1/2 inhibitor (PD) [32]. All lines (OiPS3, OiPS6, OiPS8, and OiPS16) successfully differentiated into neurons expressing the neuronal-associated proteins PAX6, NESTIN, and TUJ-1, in a similar fashion to the hESC lines H1 and H9 (Figure 4A). These results are in agreement with a recent study showing that karyotype variations within iPSCs obtained from elderly subjects did not affect their capacity to differentiate into functional motor neurons [14].

The state of X-chromosome inactivation (XCI) was assessed in the four iPSC lines and compared to that of the female hESC line H9. In agreement with previous findings [33], we detected a varied XCI status in all female pluripotent stem cells, with a subset of cells clearly expressing an inactive X-chromosome (Xi) (Figure 4B, white arrows). The XCI pattern in iPSCs obtained from the 84-year-old woman was similar to that exhibited by the embryonic stem cell line H9, thus suggesting that donor age and chromosomal abnormalities do not influence the impaired ability of human pluripotent stem cells to complete Xi-reactivation under normoxic conditions [16].

Telomerase, the enzyme responsible for telomere elongation, is active in stem cells and germ cells but normally inactive in somatic cells [34]. Cellular reprogramming has been found able to restore telomerase activity in young human healthy somatic cells [1], in mouse fibroblasts from old donors [35], and in human fibroblasts obtained from patients affected by dyskeratosis congenita (DC), a disease affecting telomere maintenance causing premature aging [36]. Thus, we asked whether re-activation of telomerase could also occur in human somatic cells derived from an elderly subject. Indeed, telomerase activity was remarkably elevated in all aged-iPSC lines compared to the parental NFH2 fibroblasts and to other fibroblast cells derived from young and aged individuals (Figure 4C). Importantly, the level of telomere activation in the old donor-derived iPSCs was comparable to that of hESCs and young donor-derived iPSCs, implying that aging and karyotype alterations do not impair the reprogramming-induced restoration of telomerase activity.

### Aged-iPSCs with karyotype abnormalities acquire hESC-like mitochondrial morphology and functionality

Ultra-structural studies showed that mitochondria exhibit a typical configuration within hESCs, with round-shaped morphology and poorly developed cristae [37]. Similar features have been recently observed in mouse iPSCs [38] and human iPSCs [25], [26]. We thus aimed to investigate whether the same could hold true for iPSC lines obtained from somatic fibroblasts from very old individuals.

**Figure 5:**
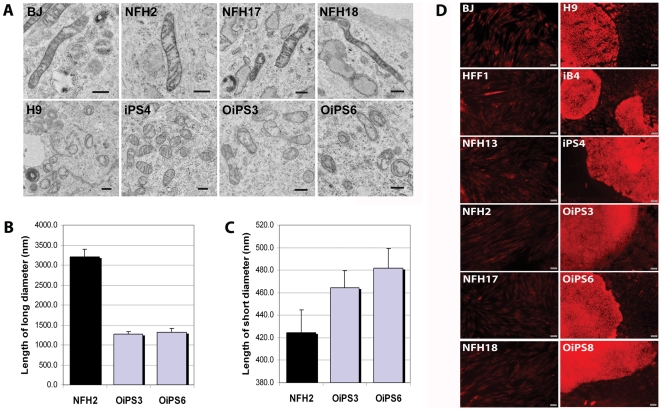
iPSCs from an 84-year-old woman acquired embryonic-like mitochondrial ultra-structure and functionality. (**A**) Transmission electron microscopy (TEM) analysis showing mitochondrial ultra-structural morphology in fibroblasts and in pluripotent stem cells. HFF1, neonatal fibroblasts; NFH2, dermal fibroblasts from an 84-year-old woman; NFH17 from an 80-year-old man; NFH18 from an 80-year-old woman; H9, female hESC line; iPS4, iPSC line derived from HFF1 fibroblasts; OiPS3 and OiPS6, iPSC lines generated from NFH2 fibroblasts. Scale bars, 500 nm. (**B–C**) Measurement of long and short mitochondrial diameters in NFH2 fibroblasts and in two NFH2-derived iPSC lines (OiPS3 and OiPS6). 50 mitochondria were measured for each sample using the EMMENU4 software (Fastscan, TVIPS). Upon reprogramming the long diameter becomes shorter while the short diameter increases, suggesting that the organelles tend to acquire a round-like shape. Error bars indicate the standard error of means (SEM). (**D**) Mitochondrial membrane potential (MMP) was assessed employing the fluorescent dye TMRE in live cells to obtain a relative measurement of mitochondria functionality in terms of energy coupling. All human fibroblasts exhibited low basal MMP. The fluorescent intensity appeared increased in undifferentiated hESCs and iPSCs, while differentiated cells at the borders of pluripotent stem cell colonies showed reduction of basal MMP signal. Thus, the mitochondrial functionality of aged iPSCs appeared similar of that of young iPSCs and hESCs. The presence of mitochondrial hyper-polarization, suggestive of reduced ATP consumption, in all pluripotent stem cells is in agreement with the distinctive metabolic features of undifferentiated stem cells which rely more on glycolytic rather than oxidative-based energy generation. Scale bars, 50 µm.

Transmission electron microscopy (TEM) images revealed that mitochondria in all fibroblast cells possess an overall comparable morphology, characterized by tubular shape and densely-packed cristae (Figure 5A). This was observed in both neonatal fibroblasts (HFF1) and in three aged-derived fibroblasts: NFH2 from an 84-year-old woman, NFH17 from an 80-year-old man, and NFH18 from an 80-year-old woman (see Table S1 for clinical details). On the other hand, the female hESC line H9 and the HFF1-derived iPSC line iPS4 showed a round-shaped morphology with sparse cristae, in agreement with previous results [25], [26]. Remarkably, the same ultra-structural traits could be identified in NFH2-derived iPSC lines OiPS3 and OiPS6. Quantitative measurement of mitochondrial diameters confirmed that both H9 and NFH2-iPSCs exhibit a decreased long diameter and increased short diameter, indicative that the organelles' structural morphology changes from tubular to round-shaped (Figure 5B and 5C). Thus, cellular reprogramming can overcome the age limitations to reset the mitochondrial morphology of somatic cells to an embryonic-like state, an ability that is not tainted by the presence of genomic alterations.

To assess mitochondrial functionality of aged reprogrammed cells, we analyzed the relative mitochondrial membrane potential (MMP), which is an important parameter of mitochondrial function, membrane integrity, and energy coupling. It has been recently demonstrated that MMP is elevated in undifferentiated human ESCs [39] and iPSCs [24], it decreases upon differentiation [24], and it becomes higher in MEFs undergoing the reprogramming process [40]. This pluripotency-associated mitochondrial hyper-polarization is believed to be a sign of reduced ATP consumption, which is indicative of glycolysis-based energy metabolism. Indeed, a switch from oxidative to glycolytic metabolism has been demonstrated in both human iPSCs [10], [26], [41] and murine iPSCs [40]. In agreement with these findings, we observed that MMP was low in all fibroblast cells, but increased in aged-iPSC lines OiPS3, OiPS6, and OiPS8, in a similar fashion to young iPSC lines iB4 and iPS4 and to hESC line H9 (Figure 5D). Moreover, spontaneously differentiated cells at the borders of all pluripotent stem cell colonies displayed lower mitochondrial membrane fluorescence (Figure 5D). Importantly, the re-establishment of hESC-like mitochondrial functionality was also confirmed in two additional aged-iPSC lines (Figure S5), which were generated from dermal fibroblasts derived from an unrelated 82-year-old woman (Hossini et al, unpublished).

Overall, mitochondrial energy-coupling and functionality appeared restored in aged iPSCs to a status similar to that of young iPSCs and hESCs, which is in accordance with the findings related to the ultra-structural mitochondrial morphology. Hence, the occurrence of genomic aberrations within aged reprogrammed cells did not negatively affect the reprogramming-induced mitochondrial and metabolic modulation.

### Chromosomal abnormalities within aged-iPSCs do not influence the re-establishment of apoptosis signaling

Apoptosis play a pivotal role in oncogenic transformation. Hence, we sought to investigate whether the presence of chromosomal aberrations could have a tumorigenic effect by altering apoptotic responses. Apoptosis was detected both at the basal level and upon treatment with two different concentrations (10^−9^ M and 10^−7^ M) of the cytostatic agent actinomycin D (AM), which causes cell cycle arrest and induces apoptotic cell death, in young and aged-derived fibroblasts, hESCs, and young and aged-derived iPSCs.

**Figure 6:**
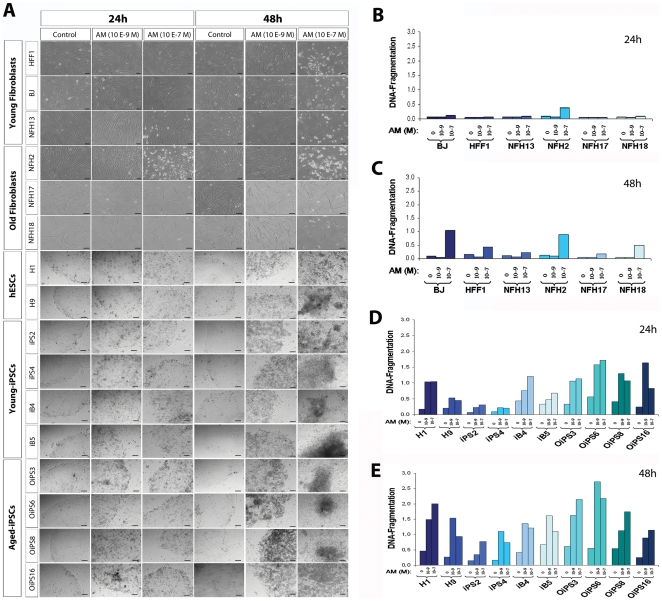
Sensitivity to drug-induced apoptosis in young and aged fibroblasts, hESCs, young-iPSCs, and aged-iPSCs. (**A**) Apoptotic cell death was measured both at the basal level and upon the treatment with two different doses of actinomycin D (AM) (10^−9^ M and 10^−7^ M). Pictures were taken after 24 h and 48 h. Scale bars, 100 µm. (**B**) Induction of apoptosis after 24 h AM treatment in young and aged-derived fibroblasts. (**C**) Apoptosis levels after 48 h AM treatment in young and aged-derived fibroblasts. (**D**) Apoptotic cell death at 24 h in hESCs, young-iPSCs (iPS2, iPS4, iB4, iB5) and aged-iPSCs (OiPS3, OiPS6, OiPS8, OiPS16). (**E**) Induction of apoptosis after 48 h AM treatment in pluripotent stem cells.

All fibroblast cells exhibited similar induction of apoptosis. Although NFH2 fibroblasts showed the presence of cell death after 24 h treatment with the highest concentration of AM (Figure 6A and 6B), all the other young and aged-derived fibroblasts started inhibiting cell proliferation activity only upon 48 h exposure to high doses of AM (Figure 6A and 6C). Conversely, pluripotent stem cells showed higher basal apoptosis activity and increased sensitivity to drug-induced apoptotic cell death. After a 24 h treatment with low doses of AM (10^−9^ M), hESCs, young-iPSCs, and aged-iPSCs exhibited a striking induction of apoptosis (Figure 6A and 6D), which further increased after 48 h (Figure 6A and 6E). Importantly, although one aged-iPSC line (OiPS6) showed slightly higher apoptotic levels than the other pluripotent stem cells, the remaining aged donor-derived iPSCs exhibited an overall susceptibility to actinomycin D-induced apoptosis similar to that of hESCs and young donor-derived iPSCs.

Transcriptional analysis of the status of the apoptotic pathway indicated the consensual up-regulation of several apoptosis-related genes in all pluripotent stem cells compared to somatic fibroblasts, these include *BIK*, *CASP3*, *HRK*, *TNF*, *TNFRSF10A-21-25*, and *TRAF2-4* (Figure S6). Taken together, all pluripotent stem cells analyzed possessed a similar hypersensitivity to cytostatic agent-induced apoptosis. Hence, karyotype variations within aged donor-derived iPSC lines might not predispose to cancer by altering the reprogramming-induced modulation of programmed cell death.

### Reprogramming endows aged cells with the ability to maintain low levels of ROS and DNA damage despite the presence of genomic aberrations

We previously demonstrated that cellular reprogramming restores a cellular state characterized by low oxidative stress levels [26]. Independent findings corroborate these results and showed that human iPSC have similar antioxidant defenses to hESCs [24]. Here, we asked whether this resetting of the cellular redox state could also occur within iPSCs with chromosomal abnormalities which were derived from very old donors, which may bear higher levels of oxidative stress and ROS-mediated DNA damage [11].

**Figure 7:**
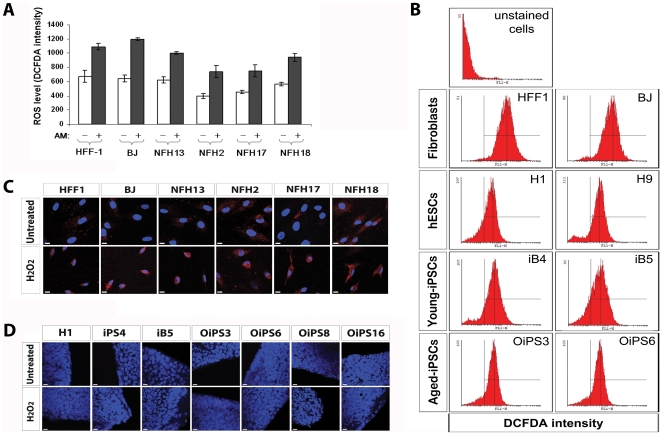
ROS and DNA damage in aged donor-derived iPSCs. (**A**) Production of ROS in fibroblast cells was measured by FACS using DCF-DA at basal level and after 24 h treatment with high doses actinomycin D (AM) (10^−6^ M). Analysis was conducted in quadruplicate and reported as fluorescence intensity. Error bars indicate standard deviation. (**B**) Representative DCF-DA-based FACS measurements of basal ROS generation in two fibroblasts (HFF1 and BJ), two hESC lines (H1 and H9), two young donor-derived iPSC lines (iB4 and iB5), and two aged donor-derived iPSC lines (OiPS3 and OiPS6). (**C**) Oxidative damage to nuclear and mitochondrial DNA within fibroblast cells was assessed by 8-hydroxy-2′-deoxyguanosine (8OHdG) staining. Treatment with 500 µM H_2_O_2_ for two hours was employed to trigger oxidative-mediated DNA damage. Scale bars, 10 µm. (**D**) Embryonic and somatic-derived pluripotent stem cells were stained with an antibody against 8OHdG to detect nuclear or mitochondrial DNA lesions induced by oxidative stress. The analysis was conducted in standard growth conditions and after a 2 h exposure to 500 µM H_2_O_2._ Scale bars, 10 µm.

Young and aged-derived fibroblasts showed an overall comparable production of ROS at basal levels and after 24 h of treatment with high doses of actinomycin D (AM) (10^−6^ M) (Figure 7A). The same AM concentration gave rise to almost complete cell death in pluripotent stem cells, due to the detected hypersensitivity to AM-induced apoptosis. However, basal levels of ROS in hESCs, young-iPSCs, and aged-iPSCs were highly reduced compared to somatic fibroblasts (Figure 7B). Importantly, the amount of ROS generated by aged donor-derived iPSCs was similar to that of young donor-derived iPSCs and hESCs.

ROS are known to oxidize DNA and indeed 8-hydroxy-2′-deoxyguanosine (8OHdG), a repair product of oxidized guanine lesions, can accumulate in both nuclear and mitochondrial DNA (mtDNA) and has been identified as a biomarker for oxidative stress [42]. The presence of cytoplasmic 8OHdG basal immunoreactivity, indicative of mtDNA lesions, was observed within fibroblasts obtained from elderly subjects (NFH2-84y, NFH17-80y, and NFH18-80y) (Figure 7C). Exposure to 500 µM of H_2_O_2_ for two hours led to increased 8OHdG signals in all fibroblasts analyzed (Figure 7C). Conversely, hESCs, young-iPSCs, and aged-iPSCs did not show signs of oxidative lesions to DNA (nuclear or mitochondrial), even after treatment with the pro-oxidant agent H_2_O_2_ (Figure 7D).

Transcriptional profiling data showed that anti-oxidant related genes such as *CAT*, *GPX1*, *SOD3* and *AOX1,* known to be involved in the response to oxidative stress, were consensually down-regulated in all pluripotent stem cells in comparison to fibroblasts, possibly due to the diminished need for counter-acting oxidative stress (Figure S7). Overall, the findings are in agreement with previously published data obtained in young donor-derived iPSCs [24], [26] and suggest that aged reprogrammed cells may be able to erase aging-associated mtDNA damage and re-establish redox homeostasis similar to that in hESCs, regardless of the presence of chromosomal aberrations.

## Discussion

Genome integrity is of critical importance for iPSC-based clinical applications. Recent findings showing the presence of nuclear and mitochondrial genomic alterations within human reprogrammed cells are causing growing concerns that tumorigenicity might ultimately hinder the clinical applicability of iPSCs [5], [6], [7], [8], [9], [10]. In order to distinguish harmless variations from those impairing the functionality or promoting clinical risks, it is then essential to investigate the biological consequences of these modifications within distinct human iPSC lines.

The detected chromosomal abnormalities have so far not been strongly linked with a functional defect. Pasi et al. observed that genomic aberrations might be transient and that they do not compromise cellular functionality [7], while Boulting et al. showed that several iPSC lines harboring karyotype variations could pass a stringent test of differentiation capacity [14]. Moreover, the presence of mitochondrial mutations within human iPSCs did not affect the reprogramming-induced modulation of bioenergetic metabolism [10]. Our current results are in agreement with these findings and further suggest that chromosomal abnormalities do not impart a negative effect on pluripotency and reprogramming-related functions, such as telomerase activity, X-chromosome activation, neuronal differentiation, and mitochondrial and metabolic re-configuration.

Oncogenic transformation is linked with a loss of apoptotic susceptibility. On the other hand, both young and aged donor-derived iPSCs and hESCs showed hypersensitivity to drug-induced apoptosis, regardless of chromosomal aberrations. This is in accordance with the high susceptibility to apoptotic cell death that pluripotent stem cells have been found to exhibit upon exposure to γ-irradiation [19]. Redox imbalance and DNA damage are additional traits of tumour development. However, the hESC and iPSC lines we tested showed low concentration of ROS and lack of oxidative damage to mitochondrial or nuclear DNA. Taken together, the data suggest that if genomic aberrations might confer oncogenic properties to pluripotent stem cells, this may not be due to the alteration of mitochondrial-associated cell death mechanisms.

Future studies will have to address whether additional cell death pathways, such as autophagy, might also be conserved in iPSCs bearing genomic variations. It is also worth pointing out that our study was conducted using retroviruses, which may cause genome aberrations within the proximity of viral integrations [43]. Thus, it will be important to investigate whether non-viral based reprogramming (*in vitro* mRNA, episomal plasmids or miRNA), direct conversion of fibroblasts [44], or drug-based protective measures, such as modulation of ROS homeostasis, could preserve aged somatic cells from acquiring chromosomal aberrations [45]. Finally, it is now an auspicious time for undertaking a systematic analysis to study in detail various nuclear and mitochondrial alterations, their functional effects and their specific risk for oncogenic transformation.

Induced pluripotent stem cells offer a unique system to study the cellular and molecular mechanisms underlying physiological and pathological aging. Two prominent theories regarding cellular aging involve the progressive loss of telomere function [46] and the oxidative-stress mediated mitochondrial damage [11], [47]. A link between these two mechanisms has been recently suggested, as on one hand, mitochondrial ROS could give rise to genotoxic damage and erosion of telomeres [48], and on the other, telomere damage could trigger a p53-mediated mitochondrial dysfunction [49]. Moreover, advanced telomere shortening has been detected in patients with respiratory chain dysfunction [50]. Our data support this connection and further demonstrate that both telomerase and mitochondrial activity are extensively modulated upon cellular reprogramming of aged somatic cells. Additional studies are warranted to dissect the chain of events underlying this reprogramming-induced reversal of cellular aging. This might eventually increase our meagre understanding of the aging process and possibly aid in the development of therapeutic approaches aimed to ameliorate or reverse aging-associated phenotypes.

Although our study was mainly limited to a single 84-year-old donor, the findings were comparable in four distinct iPSC lines derived from this individual. As an extension and further validation of these findings, the reconfiguration of mitochondrial functionality was confirmed in two additional iPSC lines generated from an unrelated 82-year-old subject. At present, we cannot exclude that the extent of the reprogramming-induced re-establishment of hESC-like rejuvenated features may exhibit inter-variability between individuals. Nonetheless, our data are in agreement with recent studies conducted on somatic fibroblasts obtained from a 70-year-old individual [25], [51], from old donor mice [35], and from young patients affected by premature aging disorders, such as dyskeratosis congenita [36] and Hutchinson-Gilford progeria [52], [53], consensually implying that cellular reprogramming may be capable of reversing aging-associated phenotypes.

Overall, we have shown that fibroblasts from a very old human donor can be coaxed into acquiring pluripotency and re-establishing rejuvenated properties related to telomerase activity, mitochondrial morphology and functionality, and mitochondria-related regulation of apoptosis and ROS generation, despite the presence of genomic alterations. Nevertheless, safeguarding the integrity of the genome of aged somatic cells, which may exhibit elevated susceptibility to genomic instability, will be essential before employing iPSC-based cellular therapies for age-associated disorders.

## Materials and Methods

### Ethics Statement

Adult dermal fibroblasts were obtained from full-thickness skin biopsies originating from the sun-protected inner side of the upper arm of patients undergoing surgery, after the approval of the study protocol by the Ethics committee of the Charité Universitaetsmedizin Berlin (Germany) and written informed consent.

### Cell culture conditions

Neonatal foreskin fibroblasts HFF1 and BJ were purchased from ATCC (HFF1 # SCRC-1041 and BJ # SCRC -2522). Adult dermal fibroblasts included a 56-year-old male (NFH13), an 84-year old female (NFH2), an 80-year-old male (NFH17), and an 80-year-old female (NFH18). Medical conditions of these subjects are reported in Table S1. NF46 fibroblasts were obtained from an 82-year-old woman (Hossini et al, unpublished). All fibroblast cells were cultured in DMEM supplemented with 10% fetal calf serum, nonessential amino acids, L-glutamine, penicillin/streptomycin and sodium pyruvate (all from Invitrogen, CA, USA). hESCs lines H1 and H9 were purchased from (WiCell #WA01 and #WA09, respectively). hESCs and iPSCs were cultured in hESCs media containing KO-DMEM supplemented with 20% knockout serum replacement, nonessential amino acids, L-glutamine, penicillin/streptomycin, sodium pyruvate, 0.1 mM β-mercaptoethanol (all from Invitrogen, Carlsbad, CA) and 8 ng/ml basic fibroblast growth factor (bFGF) (Prepotech, Rocky Hill, NJ). Cultures were maintained on mitomycin C-inactivated mouse embryonic fibroblast (MEFs) and passaged using the cut-and-paste technique. Before performing experiments, feeder-free conditions were applied and iPSCs and hESCs were grown on dishes coated with Matrigel (BD Bioscience, San Diego) in MEF-conditioned media (CM). All cultures were kept in a humidified atmosphere of 5% CO_2_ at 37°C under atmospheric oxygen conditions. Experiments were carried out with primary fibroblasts between passage 2 and passage 6 and iPSCs between passage 14 and passage 28. A summary of all cell types used in this study is reported in Table S1.

### Generation of iPS cells

HFF1-derived iPSCs (iPS2 and iPS4) and BJ-derived iPSCs (iB4 and iB5) were previously obtained using the Yamanaka retroviral cocktail [1], [10], [26]. NFH2-iPSCs were derived in a similar fashion. Briefly, pMX vector-based OCT4, KLF4, SOX2 and c-MYC retroviruses were generated using 293T cells, according to the conventional CaCl_2_ transfection protocol. 200,000 fibroblasts were used as input for reprogramming experiments. Four weeks after transduction, hESC-like colonies were manually picked and expanded for characterization.

### *In vitro* and *in vivo* differentiation

For *in vitro* differentiation, embryoid bodies (EBs) were generated from iPSCs by harvesting the cells and seeding them onto low-attachment dishes in differentiating medium without bFGF supplementation. One week later, EBs were plated onto gelatin-coated tissue culture dishes and grown for an additional ten day period. *In vivo* teratoma assays were performed by EPO-Berlin Gmbh. iPSCs were collected by trypsinization, washed and injected s.c. into NOD.Cg-Prkdcscid Il2rgtm1Wjl/SzJ mice, commonly known as NOD scid gamma (NSG). Histological analysis was performed by a certified pathologist at the Institut für Tierpathologie in Berlin, Germany.

### Neuronal differentiation

Neuronal cells were generated following a recently published adherent protocol [32]. Briefly, pluripotent stem cells were mechanically dissociated and grown in CM on matrigel-coated dishes for 72 h. Differentiation was initiated by removing bFGF and adding 10 µM SB431542 (SB) plus 1 µM PD0325901 (PD) (both from Sigma-Aldrich, Deisenhofen, Germany). Medium was changed daily and the cells were grown for additional 5 days and finally fixed for immunostaining with specific antibodies.

### Global gene expression analysis

Total RNA was quality-checked by Nanodrop analysis (Nanodrop Technologies; Wilmington, DE, USA) and a quantity of 400 ng was used as input. Biotin-labeled cRNA was produced using a linear amplification kit (Ambion; Austin, TX, USA). Hybridizations, washing, Cy3-streptavidin staining and scanning were performed on the Illumina BeadStation 500 platform (Illumina; San Diego, CA, USA), according to the manufacturer's instruction. cRNA samples were hybridized onto Illumina human-8 BeadChips version 3. The following samples were hybridized in duplicate: H1, H9, HFF1, BJ, NFH13, NFH2, NFH17, NFH18, iPS2, iPS4, iB4, iB5, OiPS3, OiPS6, OiPS8, and OiPS16. All basic expression data analysis was carried out using the BeadStudio software 3.0. Raw data were background-subtracted and normalized using the “rank invariant” algorithm and then filtered for significant expression on the basis of negative control beads. Genes were considered significantly expressed with detection p values ≤0.01. Differential expression analysis was performed using the illumina custom method; the following parameters were set to identify statistical significance: differential p values ≤0.01, fold change ratio >1.5. Heatmaps were generated with Microarray Software Suite TM4 (TMEV.bat) using the SA Biosciences Apoptosis PCR Array and Oxidative Stress and Antioxidant Defense PCR Array as input gene list (www.sabiosciences.com).

### DNA fingerprinting

Fingerprinting analysis was assayed to confirm the somatic origin of iPSCs, as previously described [26]. Briefly, genomic DNA was isolated with the FlexiGene DNA kit (Qiagen) and PCR amplified to detect genomic intervals containing variable numbers of tandem repeats (VNTR). A total of 50 ng of DNA was amplified at 94°C for 1 min, 55°C for 1 min, 72°C for 1 min for a total of 40 cycles using Dyad thermal cycler (BioRad) and then resolved on a 2.5% agarose gel.

### Transmission Electron Microscopy (TEM)

Fibroblasts and pluripotent stem cells were grown on matrigel-coated Thermanox plastic coverslips (Nalge Nunc International, Rochester, NY, USA) until 70% confluent. Cells were then fixed on coverslips with 2.5% glutaraldehyde in buffer A (50 mM sodium cacodylate, pH 7.4, 50 mM sodium chloride) for 30 min at RT. Specimens were washed 3x in the buffer A and post-fixed for 1.5 h in 0.5% osmium tetroxide in H_2_O at RT, followed by 0.1% tannic acid in 100 mM HEPES, pH 7.5 for 30 min and 2% aqueous uranyl acetate for 1.5 h. Samples were dehydrated in a graded series of ethanol, embedded in Spurr's resin (Low Viscosity Spurr Kit, Ted Pella, CA, USA) and polymerised at 60°C. Coverslips were finally removed by immersing into liquid nitrogen. Ultra-thin sections (70 nm) were counter stained with uranyl acetate and lead citrate for 20 sec. Micrographs were made with a Philips CM100 using a 1 K CCD camera. Measurement of mitochondrial diameters was performed using the EMMENU4 software (Fastscan, TVIPS).

### Immunofluorescence and alkaline phosphatase staining

For immunocytochemistry, cells were fixed with 4% paraformaldehyde (Science Services, Munich, Germany) for 20 min at RT, washed twice with phosphate-buffered saline without Ca^2+^ and Mg^2+^ (PBS) and blocked with 10% chicken serum (Vector Laboratories, Loerrach, Germany) and 0.1% Triton X-100 (Sigma-Aldrich). Nuclei were counter-stained with DAPI (200 ng/ml, Invitrogen # H357, Karsruhe, Germany). Primary antibodies included SSEA1, SSEA4, TRA-1-60 and TRA-1-81 from the ES cell characterization tool (all 1∶100, Millipore #SCR004, Schwalbach, Germany), NANOG (1∶100, Abcam #ab62734, Cambridge, UK), SMOOTH MUSCLE ACTIN (SMA) (1∶100, Dako #M0851, Hamburg, Germany), ALPHA FETO PROTEIN (AFP) (1∶100, Sigma-Aldrich #WH0000174M1), SOX17 (1∶50, R&D #AF1924, Minneapolis, MN, USA), PAX6 (1∶300, Covance #PRB-278P, Muenster, Germany), NESTIN (1∶200, Chemicon #MAB5326, Neuernberg, Germany), TUJ-1 (1∶1000, Sigma-Aldrich #T8660), BRACHYURY (T) (1∶50, R&D #AF2085), PAX6 (1∶300, Covance #PRB-278P), H3K27me3 (1∶100, Abcam #ab6002). Secondary antibodies used were conjugated with either Alexa 488 or Alexa 594 (Invitrogen #A11001, A11055, A21201, A21468, A11005, A21442). Coverslips were mounted using Dako fluorescent mounting medium (Dako #S3023) and visualized using a confocal microscope LSM 510 (Carl Zeiss, Jena, Germany). Alkaline phosphatase (AP) staining was performed following the manufacturer's instructions (Millipore #SCR004).

### Analysis of telomerase activity

Telomerase activity was determined employing the TraPEZE RT Telomerase Detection Kit (Millipore, #S7710, Temecula, CA, USA), according to the recommendations of the manufacturer. Briefly, cell pellets of the samples were resuspended in CHAPS Lysis Buffer and aliquots stored at −80°C. The protein concentrations were measured by the Bradford Assay (Bio-Rad, #500-0205, Hercules, CA, USA). Of each sample, 100 ng of total proteins were adjusted to a volume of 2 µl by CHAPS Lysis Buffer and used for the Telomerase activity assay. The quantitative Real-Time polymerase chain reactions were performed in 96-well optical reaction plates (Applied BioSystems, Foster City, CA, USA) using the ABI PRISM 7000 Sequence Detection System. Reactions were carried out in triplicates using the recommended Titanium Taq Polymerase (BD Clontech). The recommended PCR parameters were optimized by extending the last 45°C step from 10 sec to 30 sec. The following control reactions were performed: telomerase positive extract control (TPC), 100 ng of positive control cell protein extract provided by the kit adjusted to 2 µl by CHAPS Lysis Buffer, Minus telomerase Control (MTC), cell sample substituted by 2 µl CHAPS Lysis Buffer, No template Control (NTC), cell sample substituted by 2 µl water. For heat inactivation controls, protein lysates were heated at 85°C for 10 min to destroy activity of telomerase. The copy number values were calculated by converting the Ct values according to the standard curve obtained by the TSR8 Control template reactions (dilution series).

### Detection of apoptosis

After a 24 h and 48 treatment with actinomycin D (AM) (10^−9^ M and 10^−7^ M, respectively), cell apoptosis was quantified by a cell death detection enzyme-linked immunosorbent assay (ELISA) (Roche Diagnostics, Mannheim, Germany). The test detects mono- and oligonucleosomes formed in apoptotic cells according to a protocol described previously [54]. The relative apoptosis values were normalized according to cell numbers as measured by lactate dehydrogenase (LDH) (Roche Diagnostics) content in the extract buffer.

### Measurement of cellular production of reactive oxygen species (ROS)

In order to determine intracellular ROS levels, the fluorescent dye 2′,7′-dichloro-dihydro-fluorescein diacetate (DCF-DA) was used. Cells were treated with 10^−6^ M actinomycin D over 24 h and subsequently stained with 15 mM DCF-DA (Molecular Probes, Eugene, OR, USA) over 30 min, harvested by trypsinization and analyzed in PBS buffer (Biochrom, Berlin, Germany) by a FACS Canto (BD Biosciences, Heidelberg, Germany).

### Karyotyping and gene expression-based analysis of chromosomal integrity

For detection of possible karyotype abnormalities in iPSCs, chromosomal analysis after GTG-banding was performed at the Human Genetic Center of Berlin, Germany. For each line, 20 metaphases were counted and 12 karyograms were analyzed, as reported earlier [26]. Enrichment or loss of whole chromosomes or chromosomal arms was determined using the software Expander (http://acgt.cs.tau.ac.il/expander) and EASE (http://david.abcc.ncifcrf.gov/ease/ease1.htm), following a previously established approach [6]. For iPSCs, the lists of up- or down-regulated genes were obtained by comparing each iPSC line to the specific parental fibroblast cells (>2 fold change). For hESCs, their expression values were related to the average expression values of all the pluripotent stem cells included in the dataset (>2 fold change relative to grand population media). The lists of up- or down-regulated genes were then used for location enrichment analysis, Enrichment or loss of whole chromosomes or chromosomal arms was determined using the software Expander (http://acgt.cs.tau.ac.il/expander) as previously described [6]. Chromosomal enrichment/loss was considered significant when Bonferroni corrected p-values <10^−4^. Moving average plots were produced using R/Bioconductor package GenomeGraphs.

### Determination of Mitochondrial Membrane Potential (MMP)

Relative MMP was assessed in live cells by using the fluorescent dye tetratmethylrhodamine ethyl ester (TMRE, #T-669, Invitrogen, CA, USA). Human fibroblasts and undifferentiated hESCs and iPSCs were incubated with 25 nM TMRE for 30 min at 37°C, washed twice with PBS, equilibrated with pre-warmed culture media, and finally imaged using a Zeiss inverted fluorescence microscope (Zeiss Axiovert 200, Carl Zeiss, Jena, Germany) equipped with a cooled digital camera (Zeiss Axiocam, Carl Zeiss). 

### Statistical analysis

Data are expressed as mean and standard deviation. Comparisons between two groups were performed by two-tailed unpaired Student's *t* test and P values of ≤0.05 were considered statistically significant. Data were analyzed using GraphPad-Prism software (Prism 4.0, GraphPad Software, Inc.) and Windows XP Excel (Microsoft).

## Supporting Information

Figure S1 **Characterization of aged donor-derived iPSC lines. (A)** Four iPSC lines were generated from NFH2 fibroblasts from an 84-year-old woman. All lines exhibited pluripotency-associated alkaline phosphatase activity (AP) and NANOG protein expression. **(B)** All lines were negative for SSEA1, a surface marker of differentiated cells, and positive for the pluripotency-associated surface markers SSEA4, TRA1-60, and TRA1-81. Representative pictures were taken from the OiPS3 line. **(C)** DNA fingerprinting analysis confirmed the somatic origin of both young donor and aged donor-derived iPSCs.
(TIF)

Figure S2 **Transcriptional profiling of NFH2-iPSCs. (A)** Table showing all the Pearson correlation values r^2^ between all the single samples analyzed. For color coding, five distinct degrees of correlation are represented: red for r^2^ = 1, orange for 1<r^2^<0.9, yellow for 0.9<r^2^<0.8, light yellow for 0.8<r^2^<0.75, and grey for r^2^<0.75. **(B)** Scatter plot graphs showing the between the hESCs and NFH2-iPSCs (r^2^ = 0.8877), HFF1-iPSCs (r^2^ = 0.8190) and NFH2-iPSCs, and NFH2 fibroblasts and NFH2-iPSCs (r^2^ = 0.6682).
(TIF)

Figure S3 **Teratoma formation of aged-iPSCs.** *In vivo* differentiation of NFH2-iPSCs. Teratoma containing structures derivatives of the three germ layers were obtained for two lines, OiPS6 and OiPS16. The remaining two lines, OiPS3 and OiPS8, generated teratomas in which we could not identify structures characteristics of the endoderm lineage (N.D. = not detected).
(TIF)

Figure S4 **Gene expression-based chromosomal analysis in hESCs and young donor-derived iPSCs.** Significant (Bonferroni corrected p-values <10^−4^) chromosomal enrichments and chromosomal losses are indicated in red. **(A)** For hESCs, the expression values were reported to the median of all the pluripotent stem cells included in the dataset. Significantly enrichment was detected for chromosome 12 in H1, while significant loss was found for chromosomes 16, 19, and 20 in both H1 and H9. **(B)** For iPSC lines, the expression values were compared to the individual parental fibroblasts (iPS2 and iPS4 to HFF1 and iB4 and iB5 to BJ). Significantly enriched chromosomes included chromosome 16 in iPS4, chromosome 19 in iB4, and chromosomes 16 and 19 in iB5. Both iB4 and iB5 exhibited a significant chromosome 3 loss.
(TIF)

Figure S5 **Modulation of mitochondrial functionality in iPSC lines derived from an additional elderly subject.** Dermal fibroblasts from an 82-year-old woman (NFH46) were reprogrammed to pluripotency using the same viral-based protocol (Hossini et al, unpublished). Two of the generated iPSC lines (iPS5 and iPS26B) were employed for the determination of the relative membrane potential (MMP) using TMRE fluorescence. Both lines exhibited higher MMP compared to the parental fibroblasts, confirming the data obtained in the four iPSC lines derived from NFH2 fibroblasts. Scale bars, 100 µm.
(TIF)

Figure S6 **Expression of apoptosis-related genes in fibroblasts and pluripotent stem cells.** Heatmap figure depicting the status of the apoptotic pathway. Values represent the log2 ratio of the array average signal of the given gene divided by the average signal of HFF1 fibroblasts (fold change 1.5, detection p value ≤0.01, and differential p value ≤0.01). Up and down-regulated transcripts are depicted in red and green respectively.
(TIF)

Figure S7 **Expression of pluripotency-associated genes and genes involved in the response to oxidative stress.** Heatmap figures showing representative pluripotency-associated genes and genes related to antioxidant response. Values represent the log2 ratio of the array average signal of the given gene divided by the average signal of HFF1 fibroblasts (fold change 1.5, detection p value ≤0.01, and differential p value ≤0.01). Up and down-regulated transcripts are depicted in red and green respectively.
(TIF)

Table S1 **Summary of the cell types used in this study.**
(DOC)

Table S2 **Complete gene list of chromosomal enrichments and chromosomal losses in all pluripotent stem cells.**
(XLS)
