# Supplementary figures and images for: Mitochondrial-Associated Cell Death Mechanisms Are Reset to an Embryonic-Like State in Aged Donor-Derived iPS Cells Harboring Chromosomal Aberrations
Source: PLoS One. 2011 Nov 14;6(11):e27352. doi: 10.1371/journal.pone.0027352 (PMC3215709; doi:10.1371/journal.pone.0027352)

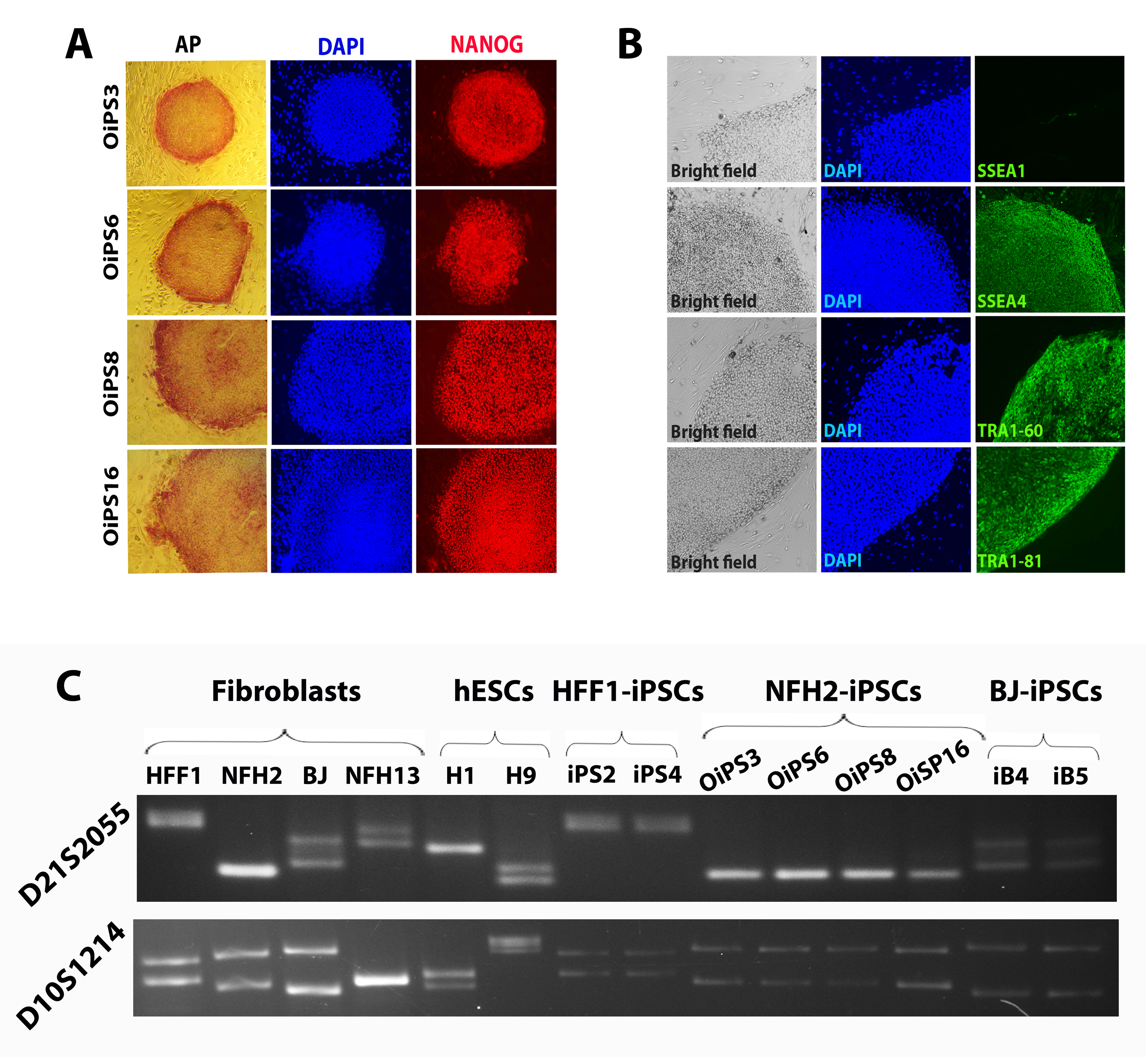

Supplement: Figure S1 — Characterization of aged donor-derived iPSC lines. (A) Four iPSC lines were generated from NFH2 fibroblasts from an 84-year-old woman. All lines exhibited pluripotency-associated alkaline phosphatase activity (AP) and NANOG protein expression. (B) All lines were negative for SSEA1, a surface marker of differentiated cells, and positive for the pluripotency-associated surface markers SSEA4, TRA1-60, and TRA1-81. Representative pictures were taken from the OiPS3 line. (C) DNA fingerprinting analysis confirmed the somatic origin of both young donor and aged donor-derived iPSCs. (TIF) [file pone.0027352.s001.tif]

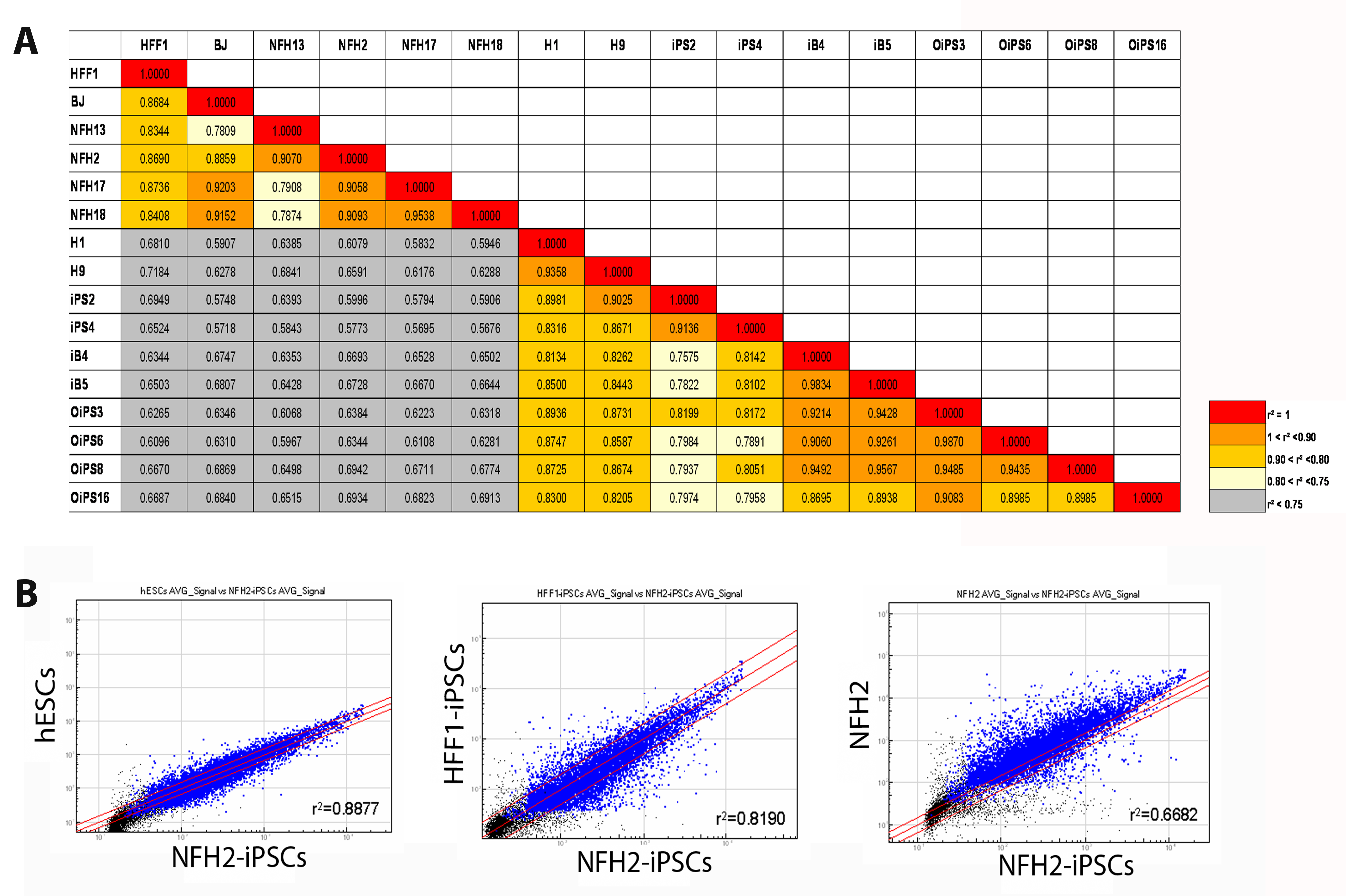

Supplement: Figure S2 — Transcriptional profiling of NFH2-iPSCs. (A) Table showing all the Pearson correlation values r2 between all the single samples analyzed. For color coding, five distinct degrees of correlation are represented: red for r2 = 1, orange for 1<r2<0.9, yellow for 0.9<r2<0.8, light yellow for 0.8<r2<0.75, and grey for r2<0.75. (B) Scatter plot graphs showing the between the hESCs and NFH2-iPSCs (r2 = 0.8877), HFF1-iPSCs (r2 = 0.8190) and NFH2-iPSCs, and NFH2 fibroblasts and NFH2-iPSCs (r2 = 0.6682). (TIF) [file pone.0027352.s002.tif]

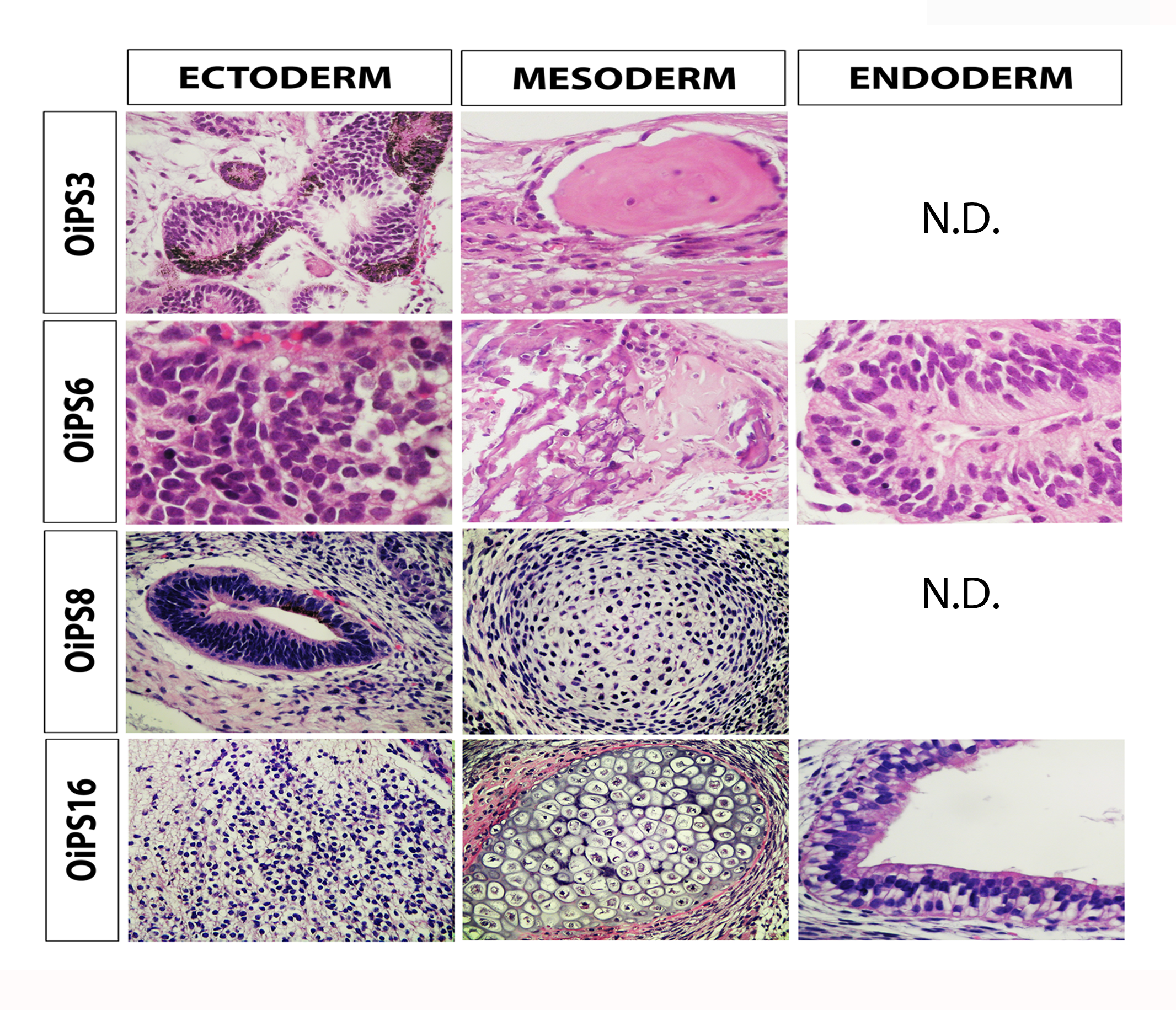

Supplement: Figure S3 — Teratoma formation of aged-iPSCs. In vivo differentiation of NFH2-iPSCs. Teratoma containing structures derivatives of the three germ layers were obtained for two lines, OiPS6 and OiPS16. The remaining two lines, OiPS3 and OiPS8, generated teratomas in which we could not identify structures characteristics of the endoderm lineage (N.D. = not detected). (TIF) [file pone.0027352.s003.tif]

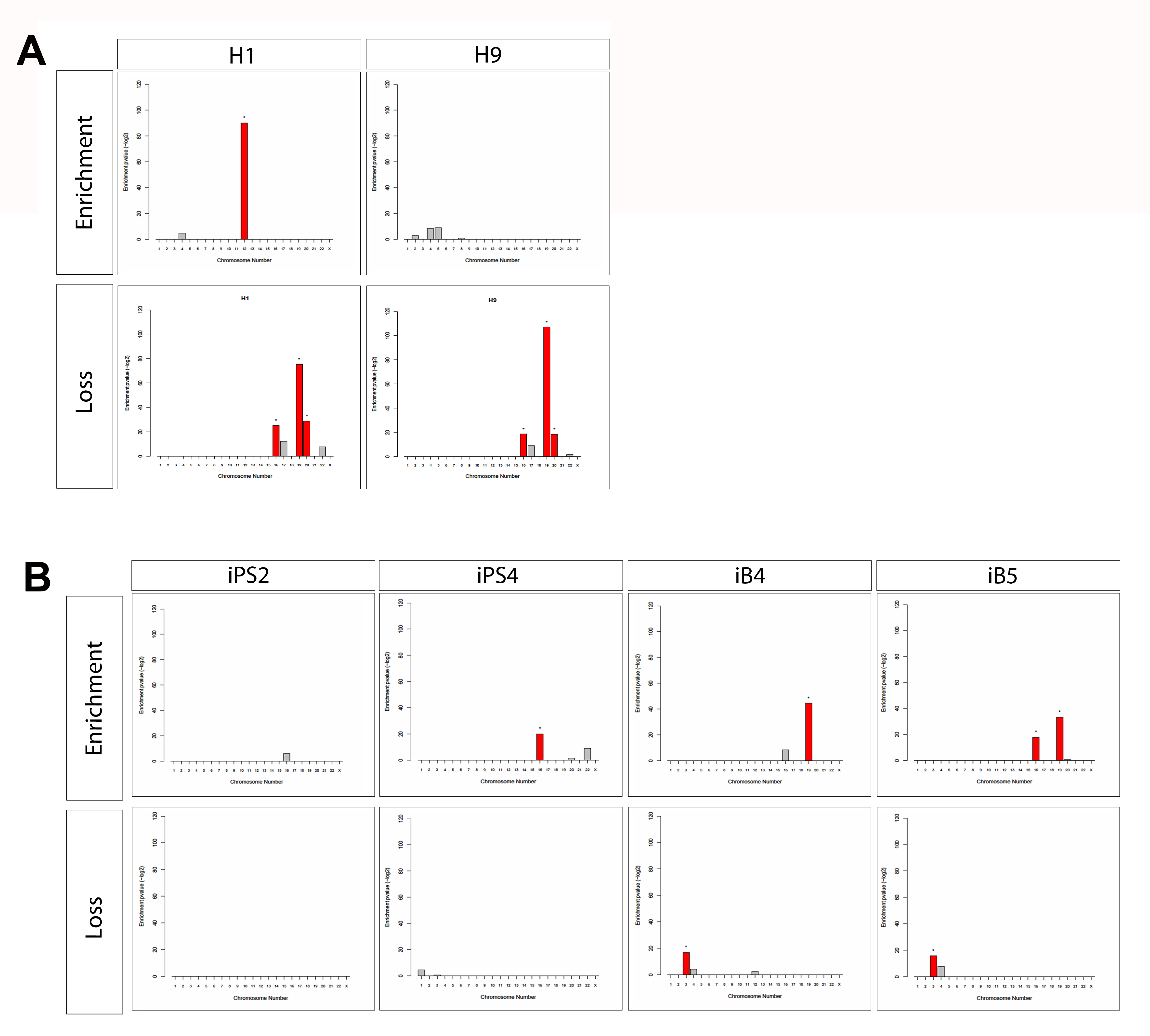

Supplement: Figure S4 — Gene expression-based chromosomal analysis in hESCs and young donor-derived iPSCs. Significant (Bonferroni corrected p-values <10−4) chromosomal enrichments and chromosomal losses are indicated in red. (A) For hESCs, the expression values were reported to the median of all the pluripotent stem cells included in the dataset. Significantly enrichment was detected for chromosome 12 in H1, while significant loss was found for chromosomes 16, 19, and 20 in both H1 and H9. (B) For iPSC lines, the expression values were compared to the individual parental fibroblasts (iPS2 and iPS4 to HFF1 and iB4 and iB5 to BJ). Significantly enriched chromosomes included chromosome 16 in iPS4, chromosome 19 in iB4, and chromosomes 16 and 19 in iB5. Both iB4 and iB5 exhibited a significant chromosome 3 loss. (TIF) [file pone.0027352.s004.tif]

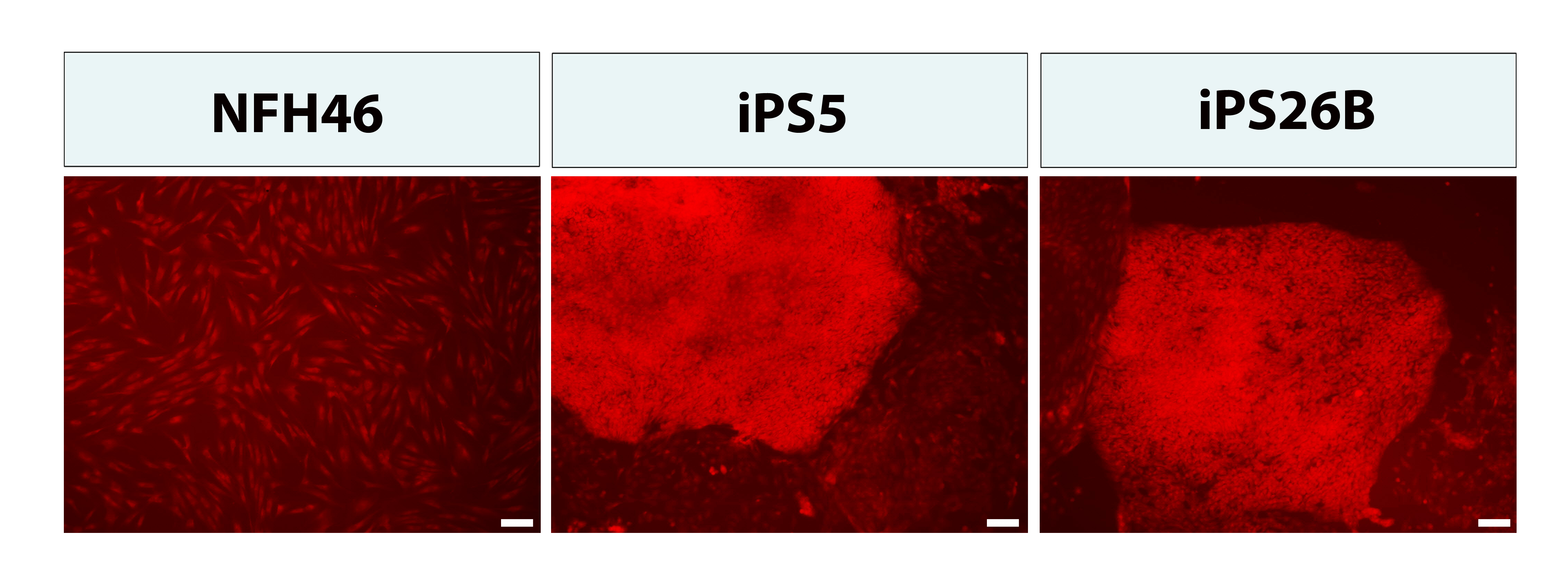

Supplement: Figure S5 — Modulation of mitochondrial functionality in iPSC lines derived from an additional elderly subject. Dermal fibroblasts from an 82-year-old woman (NFH46) were reprogrammed to pluripotency using the same viral-based protocol (Hossini et al, unpublished). Two of the generated iPSC lines (iPS5 and iPS26B) were employed for the determination of the relative membrane potential (MMP) using TMRE fluorescence. Both lines exhibited higher MMP compared to the parental fibroblasts, confirming the data obtained in the four iPSC lines derived from NFH2 fibroblasts. Scale bars, 100 µm. (TIF) [file pone.0027352.s005.tif]

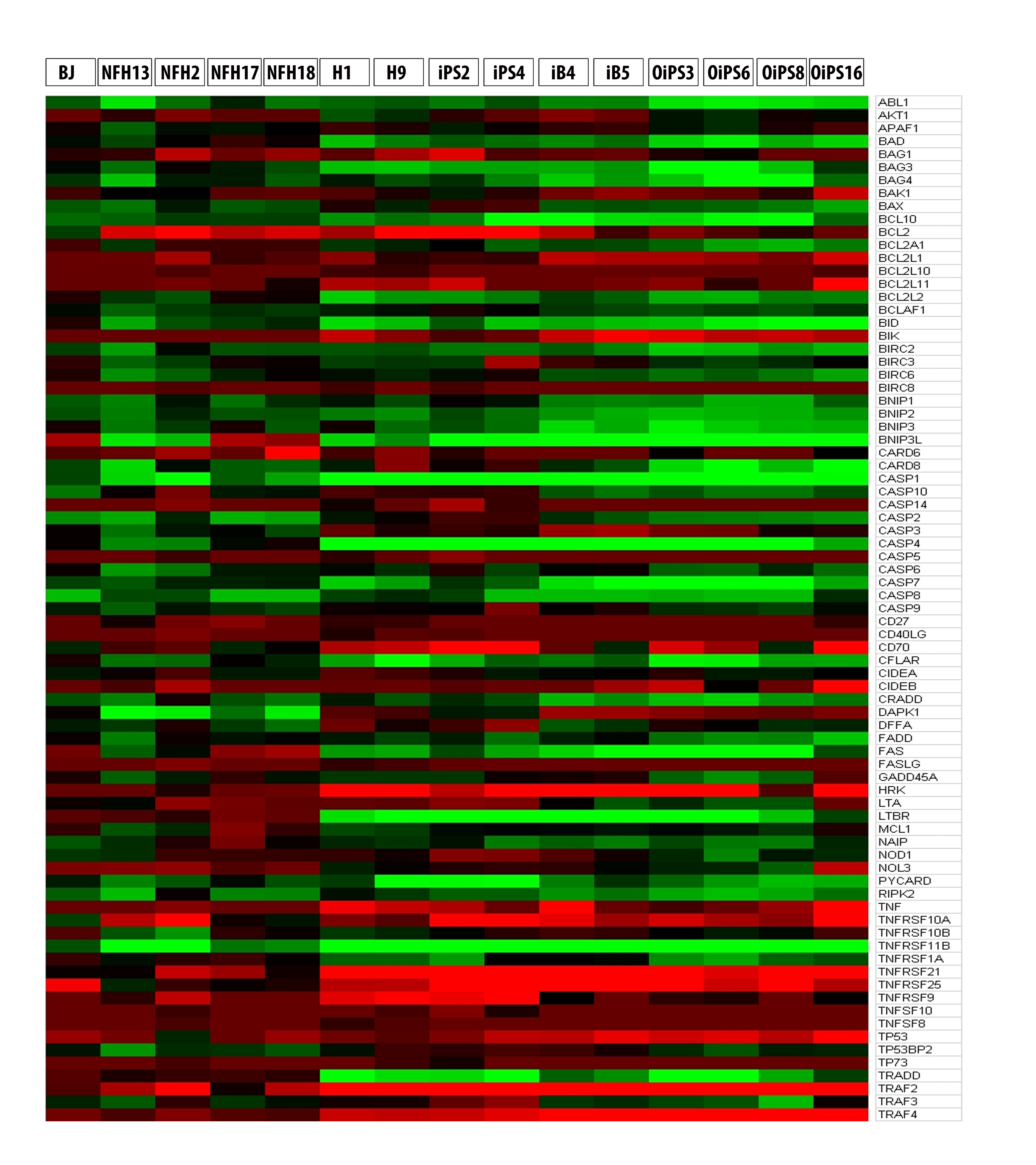

Supplement: Figure S6 — Expression of apoptosis-related genes in fibroblasts and pluripotent stem cells. Heatmap figure depicting the status of the apoptotic pathway. Values represent the log2 ratio of the array average signal of the given gene divided by the average signal of HFF1 fibroblasts (fold change 1.5, detection p value ≤0.01, and differential p value ≤0.01). Up and down-regulated transcripts are depicted in red and green respectively. (TIF) [file pone.0027352.s006.tif]

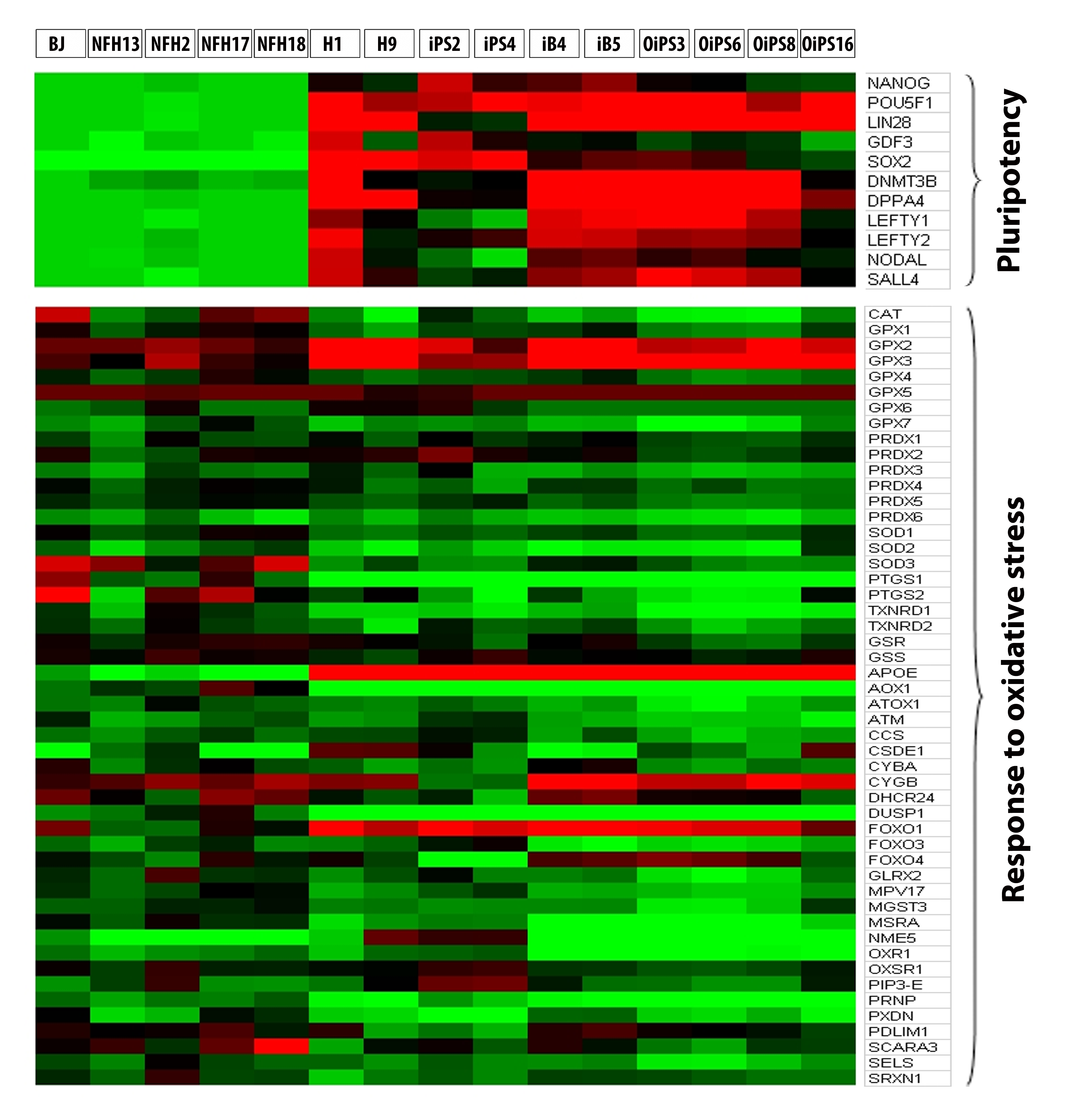

Supplement: Figure S7 — Expression of pluripotency-associated genes and genes involved in the response to oxidative stress. Heatmap figures showing representative pluripotency-associated genes and genes related to antioxidant response. Values represent the log2 ratio of the array average signal of the given gene divided by the average signal of HFF1 fibroblasts (fold change 1.5, detection p value ≤0.01, and differential p value ≤0.01). Up and down-regulated transcripts are depicted in red and green respectively. (TIF) [file pone.0027352.s007.tif]
