# Supplementary material for: Mitochondrial-Associated Cell Death Mechanisms Are Reset to an Embryonic-Like State in Aged Donor-Derived iPS Cells Harboring Chromosomal Aberrations
Source: PLoS One. 2011 Nov 14;6(11):e27352. doi: 10.1371/journal.pone.0027352 (PMC3215709; doi:10.1371/journal.pone.0027352)
Supplement: Table S1 — Summary of the cell types used in this study. (DOC) [file pone.0027352.s008.doc]

**Supp. Table 1. Summary of cell types used in the study**

|  | **Name** | **Derivation** | **Age (y=years; p=passage)** | **Sex** | **Source** | **Medical conditions** |
| --- | --- | --- | --- | --- | --- | --- |
| **Fibroblasts** | HFF1 | ATCC (SCRC-1041) | Newborn | M | Foreskin | None |
|  | BJ | ATCC (SCRC-2522) | Newborn | M | Foreskin | None |
|  | NFH13 | Dessau Clinic  (04/24/2009) | 55y | M | Upper arm | - Dermatofibrosarcoma protuberans (DFSP) - Adult onset type I diabetes - Hypertension - Hyperlipidemia - Onychomycosis |
|  | NFH2 | Dessau Clinic  (01/16/2009) | 84y | F | Upper arm | - Renal failure - Hypertension - Hypothyroidism - Type II diabetes |
|  | NFH17 | Dessau Clinic  (06/25/2010) | 80y | M | Upper arm | - Hypertension |
|  | NFH18 | Dessau Clinic  (06/25/2010) | 80y | F | Upper arm | - Hypertension |
| **Pluripotent Stem Cells** | H1 | WiCell (WA01) | 40p-50p | M | Blastocyst | None |
|  | H9 | WiCell (WA09) | 40p-50p | F | Blastocyst | None |
|  | iPS2 | Prigione et al. 2010 | 12p-25p | M | HFF1 | None |
|  | iPS4 | Prigione et al. 2010 | 12p-25p | M | HFF1 | None |
|  | iB4 | Prigione et al. 2011 | 12p-25p | M | BJ | None |
|  | iB5 | Prigione et al. 2011 | 12p-25p | M | BJ | None |
|  | OiPS3 | This study | 12p-25p | F | NFH2 | See NFH2 |
|  | OiPS6 | This study | 12p-25p | F | NFH2 | See NFH2 |
|  | OiPS8 | This study | 12p-25p | F | NFH2 | See NFH2 |
|  | OiPS16 | This study | 12p-25p | F | NFH2 | See NFH2 |
